# Differentiation of light chain cardiac amyloidosis and hypertrophic cardiomyopathy by ensemble machine learning-based radiomic analysis of cardiac magnetic resonance

**DOI:** 10.1186/s13023-025-03947-2

**Published:** 2025-11-04

**Authors:** Shuyuan Zhang, Yubo Guo, Yuze Gao, Ming Wu, Shengsheng Zhuang, Xiao Li, Ting Chen, Jian Li, Zhuang Tian, Yining Wang, Shuyang Zhang

**Affiliations:** 1 Department of Cardiology, State Key Laboratory of Complex Severe and Rare Diseases, Peking Union Medical College Hospital, Chinese Academy of Medical Sciences & Peking Union Medical College, Beijing, 100730 China; 2 Department of Radiology, State Key Laboratory of Complex Severe and Rare Diseases, Peking Union Medical College Hospital, Chinese Academy of Medical Sciences and Peking Union Medical College, Beijing, 100730 China; 3 Institute for Artificial Intelligence, Department of Computer Science and Technology & BNRist, Tsinghua University, Beijing, China; 4 Department of Hematology, State Key Laboratory of Complex Severe and Rare Diseases, Peking Union Medical College Hospital, Chinese Academy of Medical Sciences and Peking Union Medical College, Beijing, China

**Keywords:** Light chain cardiac amyloidosis, Cardiac magnetic resonance, Radiomic, Ensemble machine-learning, Diagnostic performance

## Abstract

**Background:**

We aim to assess the diagnosis performance of an ensemble machine learning (ML) based radiomic analysis of multiparametric cardiac magnetic resonance (CMR) to differentiate light chain cardiac amyloidosis (AL-CA) and hypertrophic cardiomyopathy (HCM).

**Methods:**

In the development dataset, we retrospectively collected at Peking Union Medical College Hospital between January 1, 2017, and December 31, 2022, and included 84 patients with AL-CA, 63 patients with HCM, and 34 healthy controls. Radiomics features were extracted from regions of interest in the myocardium on native T1, post-contrast T1, extracellular volume (ECV), and T2 mapping. For each modal data, eight feature selection methods were used to select the top 10 important features; then, seven ML classifiers were trained with the selected features for disease classification, and the best combinations of feature selection and classifiers were chosen by the highest predictive accuracy (ACC). The predictive results of multiple ML classifiers as input to build an ensemble ML model that classified each case (AL-CA, HCM, or controls) using a“soft voting” scheme.

**Results:**

For native T1, post-contrast T1, T2 mapping, ECV, and clinical data, the best combination of feature selection and classifier is MRMR_RF, XGboost_RF, Lasso_ Lasso, Lasso_RF, and ANOVA_ XGboost, respectively. The myocardial texture and the first-order features of native T1, post-contrast T1, and T2 mapping dominated the ensemble ML model and there was only a marginal role for ventricular shape features. In the hold-out testing dataset (37 AL-CA, 21 HCM, and 14 controls), the ensemble ML model exhibited a better diagnostic value with an area under curve of 0.98 for differentiating the 3 groups.

**Conclusion:**

An ensemble ML model with competitive diagnostic accuracy was proposed to differentiate AL-CA from HCM patients and healthy controls.

**Supplementary Information:**

The online version contains supplementary material available at 10.1186/s13023-025-03947-2.

## Introduction

Cardiac amyloidosis (CA) is a restrictive cardiomyopathy caused by the extracellular deposition of proteins in the myocardium, resulting in heart failure, conduction system disease, and sudden death [1]. Light chain cardiac amyloidosis (AL-CA) is the most common type of CA, and approximately 70% of AL-CA patients are primarily cardiac. Cardiac involvement worsens the prognosis of AL-CA patients [2], and early identification of cardiac involvement is important to improve clinical outcomes. Cardiac magnetic resonance (CMR) with quantitative tissue analysis is a highly sensitive tool for detecting cardiac involvement in AL-CA. The AL-CA patients and hypertrophic cardiomyopathy (HCM) patients share similar CMR imaging features, such as the absence of typical late gadolinium enhancement (LGE) pattern or patchy LGE [3], elevated left ventricular (LV) wall thickness, etc. It is difficult to differentiate AL-CA from HCM, thus, it is important to develop a noninvasive diagnosis tool for AL-CA.

Published research and society recommendations support CMR T1 and T2 mapping providing more diagnosis and prognosis information for AL-CA [4]. A meta-analysis revealed that native T1 without requiring contrast material has similar sensitivity and specificity to LGE in differentiating AL-CA [5]. More recently, extracellular volume (ECV) has been suggested as an alternative modality for providing incremental diagnostic and prognostic information for AL-CA patients [6]. AL-CA is characterized by variable degrees of infiltration and superimposed myocardial edema, and the intrinsic properties of the myocardium can also be measured via CMR T2 mapping. A high signal on T2 mapping of the heart in AL amyloidosis compared with controls [7], and T2 mapping is also a predictor of mortality in AL-CA patients [8].

Given the recent advances in machine learning (ML) and radiomics, it is now possible to convert CMR images into high-dimensional data to objectively and quantitatively analyze the morphological characteristics of the myocardium at the tissue level. Thus, radiomic features furnish a nearly limitless supply of imaging biomarkers with potential added value over conventional CMR metrics [9–12]. We hypothesize that a radiomics analysis of the combination with T1 mapping, T2 mapping, ECV images, and clinical data provide additional diagnosis information on AL-CA. Therefore, this study evaluated an ensemble ML framework incorporating a multimodal approach to improve the early diagnosis of cardiac involvement in AL-CA patients.

## Methods

### Study population

**Fig. 1:**
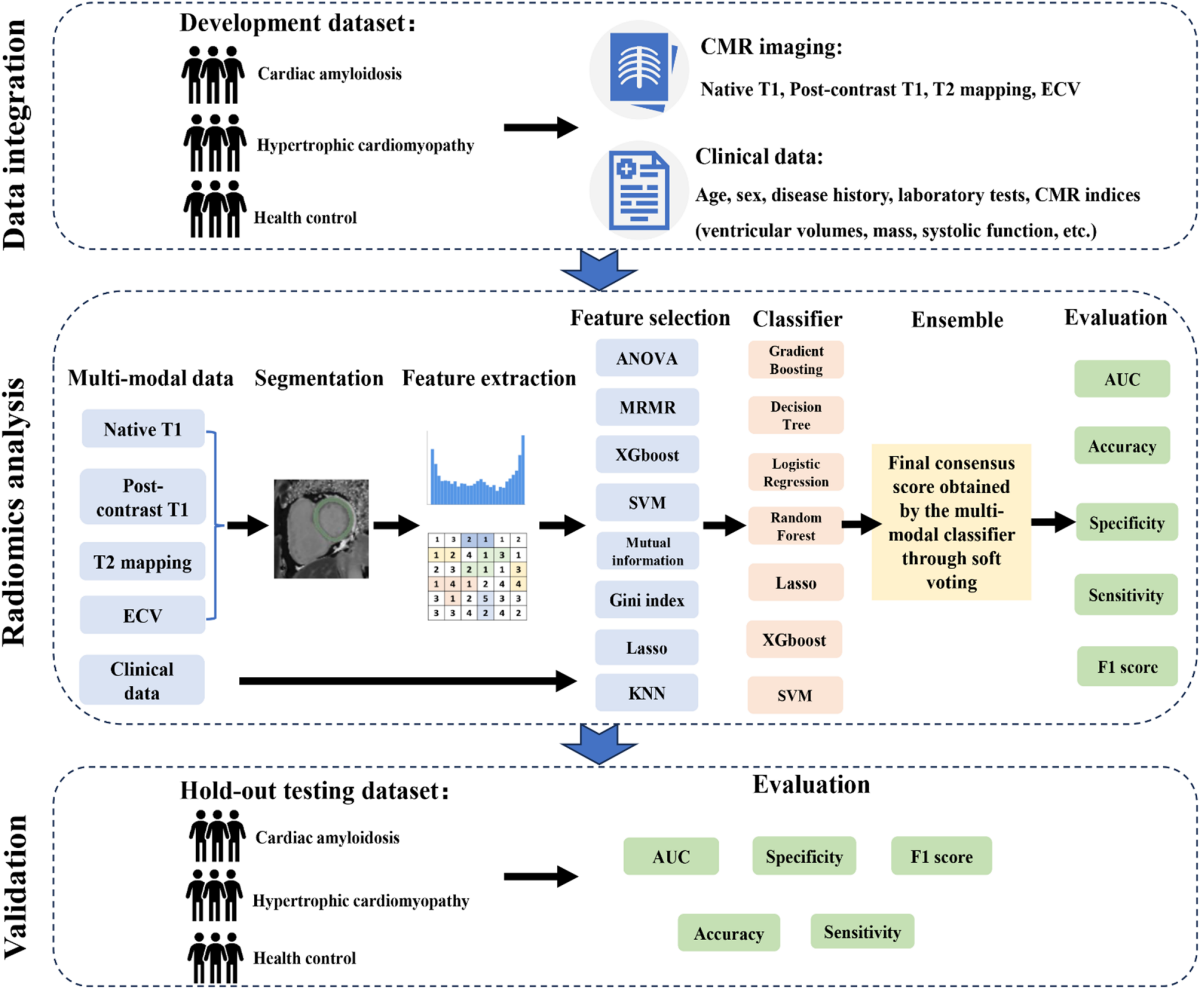
Flowchart to create the ensemble machine-learning models for the detection of cardiac amyloidosis and hypertrophic cardiomyopathy. Abbreviation: ECV, extracellular volume; CMR, cardiovascular magnetic resonance; KNN, K-Nearest Neighbor; SVM, Support Vector Machine; MRMR, Max-Relevance and Min-Redundancy; AUC, area under curve

This study consists of two independent cohorts for the development and hold-out testing datasets, respectively (Fig. 1). The development dataset was retrospectively collected at Peking Union Medical College Hospital (PUMCH) and referred for CMR imaging between January 1, 2017, and December 31, 2022, and included 84 patients with AL-CA (19 patients via extracardiac biopsy and 65 patients via endomyocardial biopsy), 63 patients with HCM, and 34 healthy controls. To further assess the disease classification performance of the model, the hold-out testing dataset was prospectively collected from PUMCH between January 1, 2023, and July 31, 2023. It included 37 patients with biopsy-proven AL-CA (26 patients via extracardiac biopsy and 11 patients via endomyocardial biopsy), 21 patients with HCM, and 14 healthy controls.

The inclusion criteria for the three groups (AL-CA, HCM, and health control) were based on established diagnostic criteria and CMR measurements. AL-CA was clinically diagnosed according to the following criteria: (1) endomyocardial biopsy (EMB) demonstrates amyloid deposits after Congo red staining, or (2) positive extracardiac biopsy combined with typical cardiac imaging features (e.g., LV wall thickness > 12 mm, relative apical sparing of global LS ratio [average of apical LS/average of combined mid + basal LS > 1], or ≥ Grade 2 diastolic dysfunction in echocardiography), or abnormal cardiac biomarkers (abnormal age-adjusted N-terminal pro-B-type natriuretic peptide [NT-proBNP]) with all other causes for these cardiac manifestations, including hypertension, reasonably excluded [2, 13]. HCM was diagnosed as follows: maximal LV thickness on CMR images ≥ 15 mm without abnormal loading conditions, or genetic testing confirmed HCM [14]. Patients with ischemic heart disease, structural heart disease, valvular heart disease, or another cardiomyopathy causing myocardial hypertrophy (such as metabolic cardiomyopathy and Fabry disease) were excluded [14, 15]. The healthy control group included participants with neither a history nor symptoms of cardiovascular disease, a negative CMR examination, and age ≥ 18 years old. Patients were excluded if they had poor-quality CMR images and/or inadequate visualization of the left ventricle (*n* = 3).

The ethical review board of PUMCH approved the study protocol, Chinese Academy of Medical Sciences, Beijing, China (protocol code I-23PJ208). All patients in PUMCH gave written informed consent. The methods are reported in line with relevant aspects of the “Radiomics Quality Score” (Table S1) [16], in which the score higher represented a higher quality of the radiomic study.

### Image acquisition protocols

**Fig. 2:**
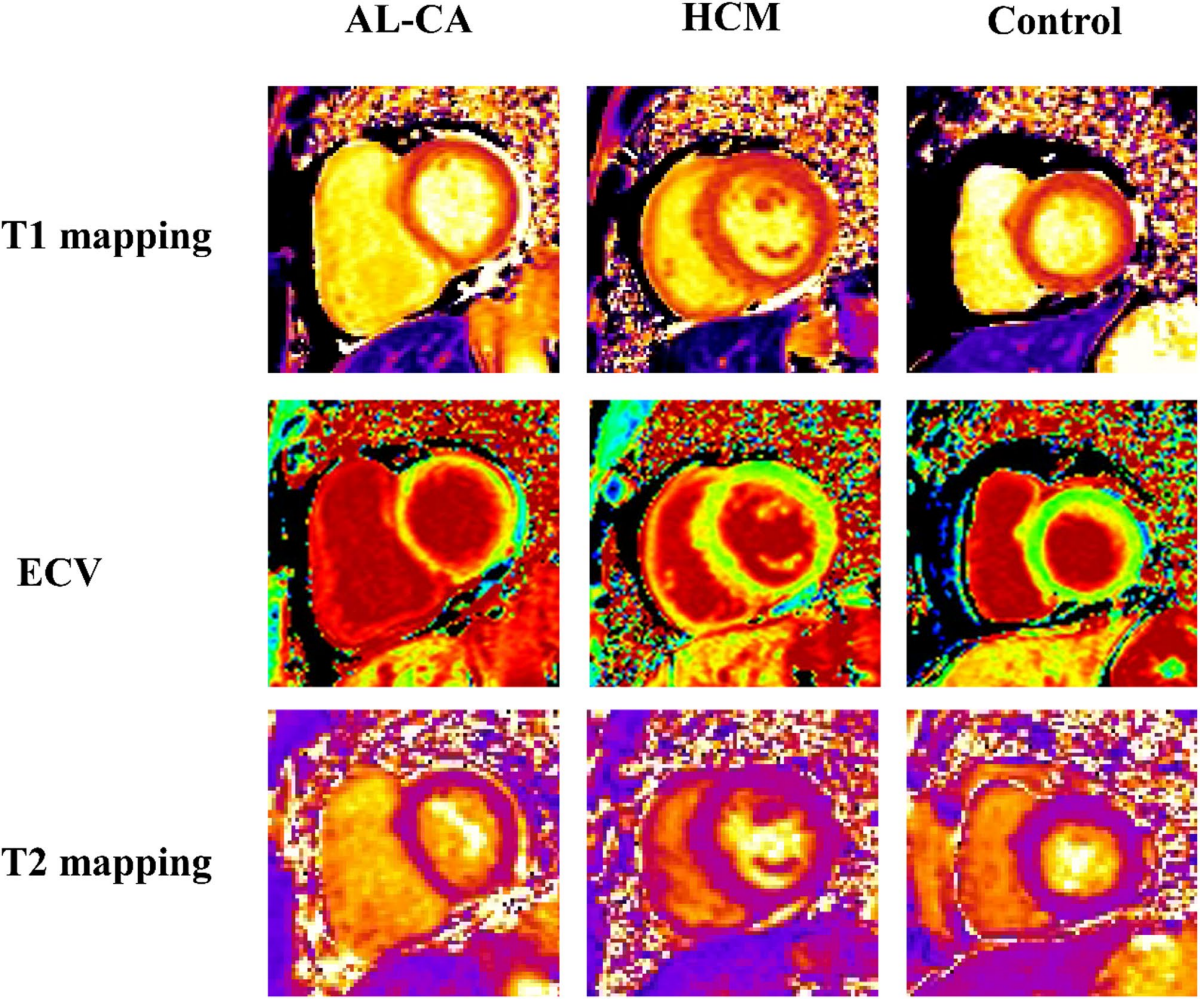
The example showed T1 mapping, ECV, and T2 mapping images for cardiac amyloidosis, hypertrophic cardiomyopathy, and healthy control in this study. Abbreviations: AL-CA, cardiac light-chain amyloidosis; HCM, hypertrophic cardiomyopathy; ECV, extracellular volume

A 3.0-T whole-body scanner (MAGNETOM Skyra, Siemens Healthineers, Erlangen, Germany) with an 18-element body matrix coil and 32-element spine array coil was used to perform CMR images. CMR T1 mapping and T2 mapping were used for each participant (Fig. 2). Native T1 and post-contrast T1 mapping, which was performed 15–20 min after intravenous gadolinium injection, were acquired at the same imaging locations using a modified Look-Locker inversion-recovery sequence, including a four-chamber long-axis view and basal-, mid-, and apical-ventricular short-axis views (repetition time/echo time/flip angle, 2.7 ms/1.12 ms/20°; voxel size, 1.4 × 1.4 × 8.0 mm^3^). Acquisition schemas 5s(3s)3s and 4s(1s)3s(1s)2s were used for pre-contrast and post-contrast T1 mapping, respectively. T2 mapping was performed using a T2-prepared single-shot bSSFP sequence with slice positions matching the T1 mapping images (repetition time/echo time/flip angle, 2.4 ms/1.0 ms/70°; field of view, 320–340 × 262–278 mm^2^; slice thickness, 8 mm; bandwidth, 1093 Hz/pixel; GeneRalized Autocalibrating Partially Parallel Acquisitions acceleration factor, 2). LGE images were collected using a phase-sensitive inversion recovery gradient-echo pulse sequence (repetition time/echo time/flip angle, 5.2 ms/1.96 ms/20°; voxel size, 1.4 × 1.4 × 8.0 mm^3^ ) in the same views as the cine images 10 min after intravenous gadolinium injection (0.15 mmol/kg body weight).

All echocardiographic examinations were performed using an ultrasound system (Vivid 9, GE Medical System, Milwaukee, Wisconsin). Images were obtained by the recording of at least 3 consecutive cardiac cycles at a frame rate of ≥ 60 frames/s. According to echocardiography, left atrial diameter (LAD), left ventricular end-diastolic dimension (LVEDD), left ventricular end-systolic diameter (LVESD), and left ventricular posterior wall (LVPW), and right ventricular internal dimension (RVD) were recorded.

### Cardiac magnetic resonance imaging analysis

All CMR images were transferred to an offline workstation with commercial postprocessing software Argus (cvi42 version 5.3; Circle Cardiovascular Imaging, Calgary, Canada) for blinded analysis. CMR analysis was performed by a radiologist (X.L with 8 years of CMR experience) who was blinded to the patient’s clinical data. Inter- and intraobserver variability were assessed in 20 randomly selected patients, such that one observer (M.W) performed one measurement, and a second observer (Y.B.G) blinded to the first observer’s results performed measurements at two time points at least 1 week apart. The following conventional CMR indices related to cardiac function were also considered during our analysis: LV end-diastolic volume index (LVEDVI), right ventricular (RV) end-diastolic volume index, LV ejection fraction (LVEF), RV ejection fraction (RVEF) and interventricular septum (IVS). The radiologist (X.L) visually estimated LGE in short- and long-axis views, and patients were classified as LGE (+) if they had myocardial enhancement in at least one segment. We used synthentic ECV as an alternative method for ECV quantification, removing the need for blood Hct measurement by utilizing the linear relationship between longitudinal relaxation rate (R1 = 1/T1) of blood and laboratory-measured Hct [17–19]. Details were described in the Supplemental Methods.

### Feature extraction

Regions of interest (ROIs) in the myocardium included the area between the endocardium and epicardium, defined by contours from the basal to the apical slices of the short axial LV myocardium using 3D Slicer (version 5.4.0). LV myocardium was manually segmented with the offset of epicardial and endocardial layers to avoid the involvement of the epicardial fat and blood pool, respectively. A radiologist (Y.B.G) with four years of experience in CMR manually delineated the LV myocardium, and then two operators (W.M. and S.S.Z) reviewed and corrected the ROIs if needed. In total, 876 triplets of slice-level CMR images were included in the development and validation datasets (Table S2).

Radiomic features from each ROI were automatically computed by the open-source “Pyradiomics package (version 3.1.0)”. Radiomics features, included 18 first-order statistics, 10 shape-based, and 75 texture features (Table S3). Details were described in the Supplemental Methods, and a total of 1688 myocardial features were extracted. To reduce the dimensionality in our feature selection data set, we assessed the intra- and inter-observer reproducibility using 20 randomly selected cases. Features with an intra- and inter-class correlation coefficient < 0.80 were excluded from further analysis.

### Feature selection and model construction

In this study, extracted radiomic features for multiparametric CMR imaging and clinical features most relevant for the differentiation between AL-CA and HCM were selected for further model construction. Figure 1 shows an overview of the ensemble ML analysis. Briefly, eight feature selection methods were used to select important features and seven ML algorithms were then applied for disease classification, and the best combinations of feature selection and ML algorithms were chosen by the highest predictive accuracy (ACC). For each modal data, eight feature selection methods were implemented for a preliminary selection, and the top 10 important features were selected. The eight feature selection methods including analysis of variance (ANOVA), mutual information (MI), max-relevance and min-redundancy (MRMR), Gini index, eXtreme Gradient Boost (XGBoost), least absolute shrinkage and selection operator (LASSO), support vector machine (SVM) combined with sequential forward selection (SFS), and K-Nearest Neighbor (KNN) combined with SFS. Seven interpretable ML algorithms were investigated: XGBoost, SVM, Lasso, random forest (RF), logistic regression (LR), decision tree (DT), and gradient boosting (GB). The predictive results of multiple ML classifiers as input to build an ensemble ML model that classified each case (CA, HCM, or healthy control) using a“soft voting” scheme, and the weight of each modal data was calculated based on the area under the curve (AUC) score (Table S4). Details were present in Supplemental Methods.

We assessed the discrimination abilities of the ML models using both receiver operating characteristic (ROC) analysis with the AUC and ACC. The macro-averaged AUCs were used to evaluate the three class classifications, and macro-averaged refers to calculating the AUC corresponding to each class separately and then calculating the average AUC of all classes to obtain the final AUC. Five-fold cross-validation was also performed on all cases.

### Statistical analysis

The clinical characteristics of the three groups were compared using chi-square tests for categorical variables (expressed as numbers [percentages]), ANOVA for quantitative variables (expressed as mean ± standard deviation [SD]), and Kruskal-Wallis test for estimated glomerular filtration rate (expressed as median [interquartile range]). Statistical analyses were performed using SPSS (version 26.0, SPSS Inc., Chicago, USA). Machine-learning algorithms and feature selection methods were implemented using Python (version 3.9.13). A *P*-value < 0.05 was considered statistically significant.

## Results

### Patient characteristics

Clinical and demographic data for the development and hold-out testing dataset among AL-CA patients, HCM patients, and healthy controls are summarized in Table 1 and Table S5. It showed significant differences in clinical characteristics and CMR features among the three groups in the development dataset. Compared with HCM patients, AL-CA patients were older (58.3 years vs. 51.5 years), and significantly lower levels of CMR-measured LVEF (60.8% vs. 63.1%), RVEF (57.7% vs. 64.9%), interventricular septal thickness (13.9 ± 3.7 mm vs. 17.3 ± 3.9 mm), and LVEDVI (66.5 ± 11.7 ml/m^2^ vs. 72.2 ± 14.6 ml/m^2^). Similar results between AL-CA and HCM were found in the hold-out testing dataset.

**Table 1:** Clinical characteristics, echocardiographic and cardiac magnetic resonance features in the development dataset

Baseline Characteristics	AL-CA patients (*n* = 84)	HCM patients (*n* = 63)	Health control (*n* = 34)	*P* Value
**Clinical characteristics**				
Age, years	58.3 ± 7.8	51.5 ± 15.1*	43.6 ± 18.7**	< 0.001
Men, n (%)	48 (57.1)	36 (57.1)	18 (52.9)	0.906
SBP, mmHg	109.7 ± 15.4	128.1 ± 19.2*	125.8 ± 16.4**	< 0.001
DBP, mmHg	70.1 ± 9.8	77.1 ± 13.9*	75.3 ± 9.4**	0.001
Heart rate, bpm	84.1 ± 12.1	75.8 ± 11.6*	89.2 ± 18.4	< 0.001
NYHA cardiac function class, n (%)				0.023
I or II	64 (76.2)	53 (84.1)	33 (97.1)**	
III or IV	20 (23.8)	10 (15.9)	1 (2.9)	
Mayo stage, n (%)				
I or II	40 (47.6)	-	-	-
III or IV	44 (52.4)	-	-	
Hypertension, n (%)	12 (14.3)	28 (44.4)*	5 (14.7)	< 0.001
Antihypertensive treatment, n (%)	11 (13.1)	27 (42.9)*	4 (11.8)	< 0.001
Dyslipidemia, n (%)	5 (4.8)	16 (25.4)*	3 (8.8)	0.002
Diabetes mellitus, n (%)	5 (6.0)	8 (12.7)	4 (11.8)	0.321
Serum creatinine, mg/dL	79.1 ± 27.6	77.3 ± 21.5	56.0 ± 18.9**	< 0.001
Estimated glomerular filtration rate (mL/min/1.73 m^2^)	97.6 (74.7-109.7)	94.3 (79.9-106.1)	113.6 (104.2-125.7)**	< 0.001
cTnI, µg/L	0.05 (0.02–0.12)	-	-	-
NT-proBNP, pg/mL	1729.5 (305.7–4365.0)	-	-	-
dFLC, mg/L	119.2 (37.2-385.9)	-	-	-
**Echocardiography**				
LAD (mm)	38.1 ± 6.1	40.6 ± 8.7	32.4 ± 5.9**	< 0.001
LVEDD (mm)	42.4 ± 4.3	46.3 ± 4.8*	45.2 ± 5.0**	< 0.001
LVESD (mm)	28.1 ± 4.6	27.8 ± 5.8	28.2 ± 4.2	0.930
LVPW (mm)	11.6 ± 3.1	9.9 ± 2.9*	8.1 ± 1.2**	< 0.001
RVD (mm)	21.8 ± 4.3	22.3 ± 3.3	22.2 ± 4.0	0.785
**Cardiovascular magnetic resonance**				
LVEF (%)	60.8 ± 11.4	63.1 ± 10.6*	65.1 ± 5.8**	< 0.001
RVEF (%)	57.7 ± 11.5	64.9 ± 9.4*	59.9 ± 6.6	< 0.001
LVEDVI (ml/m^2^)	66.5 ± 11.7	72.2 ± 14.6*	67.8 ± 12.6	0.037
RVEDVI (ml/m^2^)	64.9 ± 18.3	61.1 ± 15.0	68.5 ± 16.1	0.108
IVS, mm	13.9 ± 3.7	17.3 ± 3.9*	9.4 ± 1.1**	< 0.001
LGE (+), n (%)	72 (85.7)	56 (88.9)	1 (2.9)**	< 0.001

AL-CA, light chain cardiac amyloidosis; HCM, hypertrophic cardiomyopathy; SBP, systolic blood pressure; DBP, diastolic blood pressure; NYHA, New York Heart Association; cTnI, cardiac troponin I; NT-proBNP, N-terminal pro-B-type natriuretic peptide; dFLC, serum immunoglobulin free light chain difference; LAD, left atrial diameter; LVEDD, left ventricular end-diastolic diameter; LVESD, left ventricular end-systolic diameter; LVPW, left ventricular posterior wall; RVD, right ventricular internal dimension; LVEF, left ventricular ejection fraction; RVEF, right ventricular ejection fraction; LVEDVI, left ventricle end-diastolic volume index; RVEDVI, right ventricle end-diastolic volume index; IVS, interventricular septum; LGE, late gadolinium enhanced.

Data are reported as mean ± SD or number (%) as appropriate

*P* value indicates for differences across three groups

^*, **^indicates significant difference (*P* value < 0.05) between AL-CA patients and HCM patients, and AL-CA patients and health controls, respectively

### Selection of features and classifiers

**Fig. 3:**
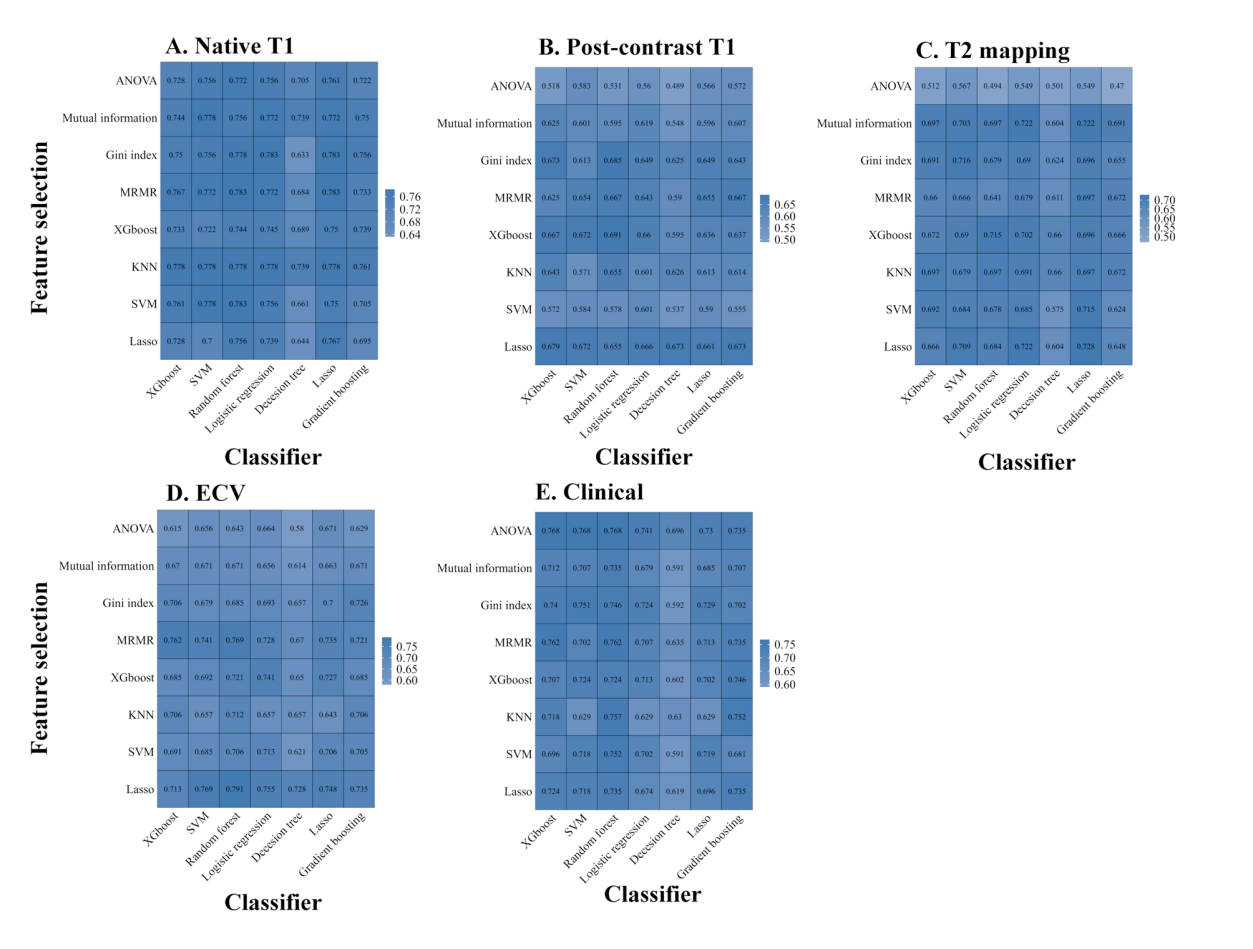
The average accuracy of feature selection and classifier for each single-modal data. Abbreviation: ANOVA, analysis of variance; MRMR, Max-Relevance and Min-Redundancy; Lasso, least absolute shrinkage and selection operator; XGboost, eXtreme Gradient Boost; SVM, support vector machine; ECV, extracellular volume

To identify the most appropriate feature selection method and classifier for each modal data, eight feature selection methods and seven classifiers were compared. For native T1, post-contrast T1 mapping, T2 mapping, ECV, and clinical data, the best combination of feature selection and classifier is MRMR_RF (the highest predictive ACC, 0.78), XGboost_RF (the highest predictive ACC, 0.69), Lasso_ Lasso (the highest predictive ACC, 0.73), Lasso_RF (the highest predictive ACC, 0.79), ANOVA_ XGboost (the highest predictive ACC, 0.77), respectively (Fig. 3). The corresponding macro-averaged AUCs for native T1, post-contrast T1 mapping, T2 mapping, ECV, and clinical data in the development dataset were 0.92 (0.88, 0.96), 0.86 (0.84, 0.88), 0.85 (0.82, 0.89), 0.91 (0.88, 0.95), and 0.90 (0.87, 0.94), respectively (Table 2).

**Table 2:** The performance of the best combination machine learning models for each modal

Modal	Combination of feature selection and classifier	AUC			Macro-averaged AUC	Accuracy	Sensitivity	Specificity	F1 score
		AL-CA	HCM	Control					
**Native T1**	MRMR_RF	0.93 (0.89, 0.96)	0.92 (0.85, 0.98)	0.90 (0.82, 0.99)	0.92 (0.88, 0.96)	0.78 (0.72, 0.85)	0.78 (0.70, 0.86)	0.89 (0.85, 0.92)	0.77 (0.69, 0.85)
**Post-contrast T1**	XGboost_RF	0.82 (0.75, 0.89)	0.83 (0.76, 0.89)	0.92 (0.88, 0.97)	0.86 (0.84, 0.88)	0.69 (0.65, 0.73)	0.67 (0.63, 0.71)	0.83 (0.80, 0.87)	0.67 (0.63, 0.71)
**T2 mapping**	Lasso_ Lasso	0.85 (0.75, 0.95)	0.85 (0.81, 0.89)	0.87 (0.75, 1.00)	0.85 (0.82, 0.89)	0.73 (0.69, 0.76)	0.72 (0.66, 0.77)	0.86 (0.84, 0.88)	0.71 (0.67, 0.76)
**ECV**	Lasso_RF	0.94 (0.90, 0.97)	0.88 (0.84, 0.92)	0.86 (0.75, 0.97)	0.91 (0.88, 0.95)	0.79 (0.72, 0.86)	0.75 (0.64, 0.87)	0.88 (0.83, 0.94)	0.75 (0.65, 0.86)
**Clinical feature**	ANOVA_ XGboost	0.91 (0.85, 0.97)	0.88 (0.82, 0.94)	0.92 (0.86, 0.97)	0.90 (0.87, 0.94)	0.77 (0.72, 0.82)	0.76 (0.70, 0.81)	0.88 (0.86, 0.90)	0.75 (0.69, 0.81)

Abbreviation: AL-CA, light chain cardiac amyloidosis; HCM, hypertrophic cardiomyopathy; AUC, area under curve; ANOVA, analysis of variance; RF, random forest; MRMR, Max-Relevance and Min-Redundancy; Lasso, least absolute shrinkage and selection operator; XGboost, eXtreme Gradient Boost; ECV, extracellular volume

### Analysis of selected features

The top 10 important features selected for each modal data and their contributions to the prediction of AL-CA are presented in Figure S1. Overall, the myocardial texture and the first-order features of native T1, post-contrast T1, and T2 mapping dominated the ensemble ML model and there was only a marginal role for ventricular shape features. Conventional CMR and echocardiography metrics were also included in the ensemble ML model for clinical data, such as IVS, LVESD, and LVPW.

### Performance of ensemble ML model

**Fig. 4:**
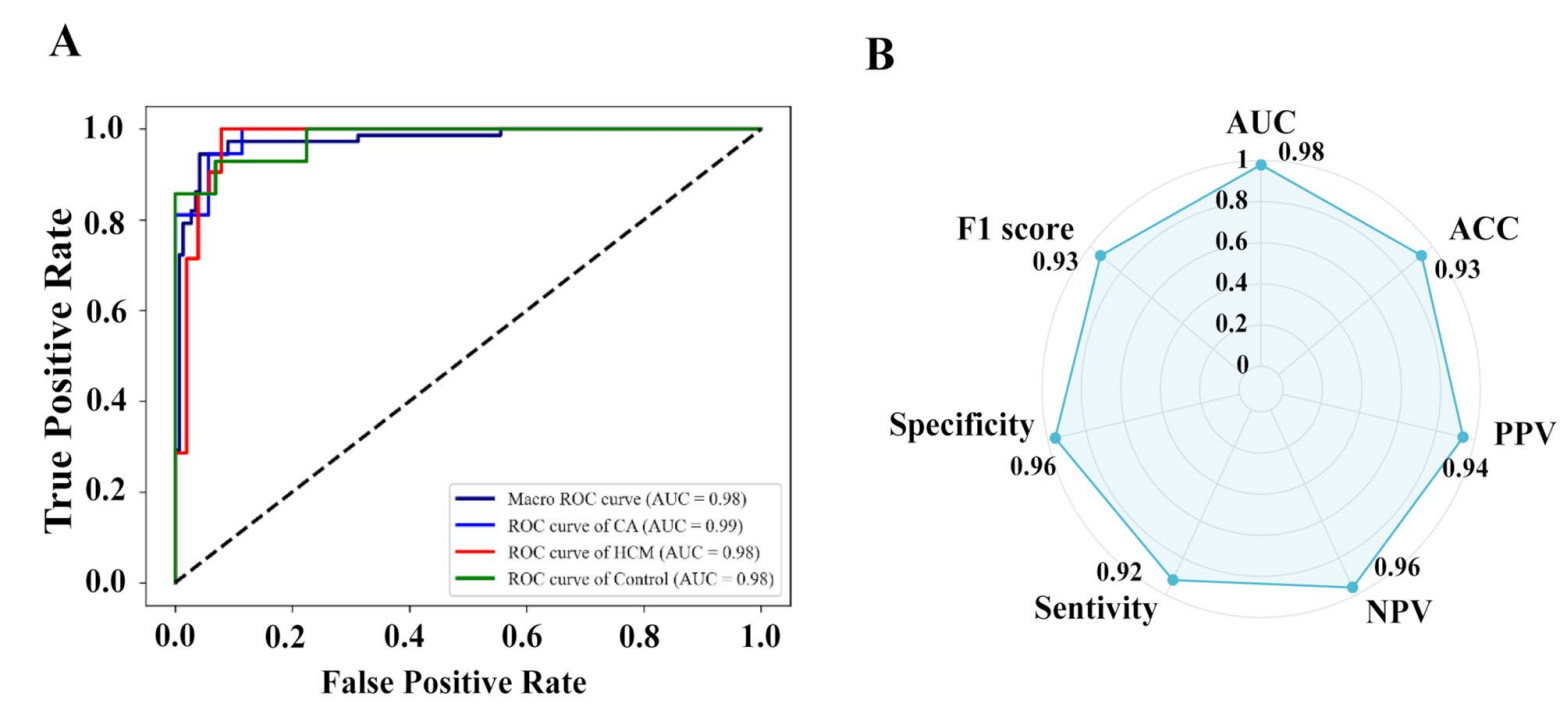
Performance of ensemble machine learning model for detection of cardiac amyloidosis and hypertrophic cardiomyopathy in the hold-out testing set. Data showed the ROC curves of the ensemble learning model for disease classification in the validation dataset (**A**); Radar plots showed the diagnostic efficacy of the ensemble learning model in the validation dataset (**B**)

Ensemble ML model is more sophisticated and diagnosed effectively than single classifiers, and merging multi-modal data with the best-performing ML models could improve the diagnostic performance. The ROC curves and AUCs of each single modal classifier in the hold-out testing dataset are shown in Figure S2. The maximum diagnostic performances (macro-averaged AUCs) of each single modal classifier were as follows: native T1 (0.89), post-contrast T1 (0.88), T2 mapping (0.89), ECV (0.91), and clinical data (0.91), respectively. We used the three classifiers random forest, lasso, and XGboost, and ensemble them using the voting ensemble method. Compared with each single classifier, the ensemble model exhibited a better diagnostic value with a macro-averaged AUC of 0.98, sensitivity of 92%, specificity of 96%, F1 score of 93%, and accuracy of 93% for differentiating the 3 groups (Fig. 4).

## Discussion

The present study demonstrated that the ensemble ML algorithms incorporating radiomics using multiparametric CMR provided high diagnostic performance for patients with LV hypertrophy phenotypes (including AL-CA and HCM). Our finding may provide a basis for developing a novel tool for AL-CA diagnosis.

Multiparametric CMR is a robust technique for diagnosis and risk stratification of patients with AL-CA in multiple studies. LGE was one of the first histologically proven methods for noninvasively detecting AL-CA, relies on a visual assessment of myocardial tissue rather than quantitative parametric mapping [20]. A meta-analysis involving five studies (*n* = 257) demonstrated that the LGE method has a high diagnostic value for AL-CA with a sensitivity of 85% and specificity of 92%, respectively [21]. The early-stage symptoms of patients with AL-CA closely resemble those of HCM. Patients with AL-CA typically present diffuse thickening of the LV wall, primarily focusing on the thickening of the ventricular septum while HCM is characterized by predominantly asymmetric thickening. However, for patients with early myocardial amyloidosis without visible thickening of the ventricular wall and hypertrophic heart patients without significant fibrosis, it is difficult to distinguish between the two based on morphology and function. A critical challenge with LGE arises in diffuse symmetrical disease, where the absence of normal myocardium as a reference precludes accurate visual detection of subtle enhancement patterns. This shortcoming underscores LGE’s semi-quantitative nature—it identifies regions of hyperenhancement relative to surrounding tissue but does not provide absolute tissue characterization. In contrast, novel CMR techniques such as T1 mapping, T2 mapping, and ECV estimation offer quantitative, parametric insights into myocardial tissue composition. These quantitative modalities allow for CA early detection and potential assessment of disease progression or remission [22]. Native T1 has emerged as a potentially useful diagnostic CMR technique for identifying AL-CA without recourse to contrast agents [23]. A meta-analysis involving 18 diagnostic studies (*n* = 2015) showed that native T1 has similar sensitivity and specificity as ECV and LGE without requiring contrast material [5]. However, in patients with only mildly elevated T1, there may be a “gray zone” where LGE or ECV is required for a more definitive diagnosis. Interestingly, our study found that the performance of the AL-CA diagnostic model using ECV (AUC = 0.91) was superior to that based on native- and post-contrast T1 mapping in the hold-out testing dataset. The amount of extracellular space in the interventricular septum estimated from native‐ and post-contrast T1 mapping showed a close relationship with the degree of extracellular matrix remodeling at histology, better represented by the sum of fibrosis and amyloidosis [24]. ECV has become a well-established surrogate for diffuse fibrosis and quantifying myocardial changes that are not visible on LGE, making it a valuable tool for the early detection of AL-CA [25]. Yue et al. also found that T1 mapping and ECV are more sensitive than LGE in detecting early myocardial involvement in AL-CA [26]. These findings supported our results that combining T1 mapping and ECV improves the performance of the AL-CA diagnostic tool.

In our study, the radiomics analysis assessing the utility of T2 mapping showed a potential diagnostic performance in AL-CA patients (in the hold-out testing dataset, AUC = 0.89). T2 mapping is a non-contrast technique, which could be applied for patients with renal impairment. Unlike T1 mapping and ECV, very few studies have evaluated the use of T2 mapping in patients with AL-CA, and these results are still controversial. A study investigating the diagnostic and prognostic utility of myocardial native T2 mapping in cardiac amyloidosis demonstrated significantly elevated native T2 among AL-CA patients compared to healthy controls, suggesting its potential as a biomarker for myocardial involvement [7]. CMR radiomics has the potential to transform our approach to defining imaging phenotypes and improve diagnostic accuracy. Previous studies have demonstrated superior diagnostic accuracy of CMR radiomics analysis compared with conventional reporting [11, 12, 27, 28]. In our research, the radiomics analysis of T2 mapping offered new insights into the early identification of myocardial involvement.

Among the radiomic features extracted from T1 mapping and T2 mapping, the most contributing were texture features compared to the first-order and shape features. Textural features describe the spatial relationship between neighboring voxels’ signal intensity quantifying myocardial heterogeneity, and the two most contributing texture features were the grey level dependence matrix (GLDM) and grey level run-length matrix (GLRLM). The former reflects the local grey level distribution and spatial dependency in the image and is used to assess the complexity and consistency of the texture in the image. The GLRLM reflects the coarseness and directionality of the texture in the image and is used to evaluate the fine details and structural complexity. The previous study found that radiomics analysis based on texture features of native T1 images discriminates between hypertensive heart disease and HCM patients providing a diagnostic accuracy of 80% [12]. Our results combined radiomics features from multiparametric CMR leading to an increment in test accuracy of 93% to differentiate CA patients, suggesting that these features reflecting the biological properties of myocardial heterogeneities, may provide more information in predicting CA diagnosis.

Our findings have some potential clinical implications. Cardiac amyloidosis is associated with poor outcomes, so early cardiac diagnosis and subsequent cardiac-directed therapy are becoming increasingly important. We used an ensemble machine-learning model to integrate multiparametric radiomics and clinical features to differentiate patients with LV hypertrophy phenotypes. The ML model integrated high-volume data generated from cardiac imaging in a quantitative modalities approach that could diagnose disease efficiently, especially in early-stage AL-CA patients without the typical LGE pattern. Combining automated diagnostic algorithms with widely available clinical imaging data can reduce physician burden while providing more opportunities for targeted cardiovascular care.

Our study had some limitations. First, we performed our radiomics model in a single center using a 3.0T CMR scanner with internal validation only; a larger multicenter, multivendor study could improve the generalizability and robustness of the results through external validation. Second, our algorithm was trained and evaluated on a cohort with specific demographic and clinical characteristics. Increased heterogeneity of our data samples will improve its generalizability. Third, we focused on the diagnosis and classification among AL-CA, HCM, and controls. Further studies could explore the utility of radiomics analysis of CMR in predicting the prognosis of AL-CA.

## Conclusion

We constructed an ensemble ML model that integrates multiparametric CMR, radiomics, and clinical features to quantitatively differentiate AL-CA from HCM patients and healthy controls. However, further studies are needed to validate the generalizability of these ensemble ML algorithms in larger populations.

## Supplementary Information

Below is the link to the electronic supplementary material.

Supplementary Material 1

## Data Availability

The authors are willing to provide a de-identified copy of the data upon reasonable request.
