# Supplementary material for: Differentiation of light chain cardiac amyloidosis and hypertrophic cardiomyopathy by ensemble machine learning-based radiomic analysis of cardiac magnetic resonance
Source: Orphanet J Rare Dis. 2025 Nov 4;20:557. doi: 10.1186/s13023-025-03947-2 (PMC12584318; doi:10.1186/s13023-025-03947-2)
Supplement: Supplementary file 1 — Supplementary Material 1 [file 13023_2025_3947_MOESM1_ESM.docx]

**Supplemental material**

**Supplemental Methods**

**Extracellular volume (ECV) measurements**

Two quantitative T1 maps generated from modified Look-Locker Inversion-recovery images acquired 15 min after (post-contrast T1) and before (native T1) administration of a gadolinium-based extracellular contrast agent. Both two T1 maps are displayed with the same grey scale. The reciprocal of each pixel value is taken to generate R1 maps (bottom-left two images labeled R1). The native T1 R1 map pixel values are subtracted from the post-contrast T1 R1 map to generate a ΔR1 map. The R1 maps and the ΔR1 map are all displayed with the same grey scale. In the ΔR1 map, the ΔR1 value of the left ventricular blood pool is measured in a region of interest (black oval). Instead of using venous hematocrit values, synthetic ECV estimation derives hematocrit from the pre-contrast blood T1 value according to the formula: Synthetic hematocrit = 869.7 × (1/T1_blood.pre_) – 0.071. The ΔR1 of the myocardial region of interest is divided by the ΔR1 of the blood pool and multiplied by one minus the synthetic hematocrit to compute pixel-wise synthetic ECV values, ranging in vales between 0 and 100%. Estimation of the synthetic ECV requires measurement of myocardial and blood T1 before and after administration of contrast agents as well as the synthetic hematocrit value according to the formula:

Synthetic ECV= (1- synthetic haematocrit) × {[(1/T1_myocardium.post_) − (1/T1_myocardium.pre_)]/[(1/T1_blood.post_) − (1/T1_blood.pre_)]} × 100% [1].

**Radiomics feature extraction**

Myocardial radiomics features, which provide a thorough morphological phenotyping of the myocardium, were extracted from images using ‘Pyradiomics’ package and divided into three main groups: (i) first-order (statistics), (ii) texture, and (iii) size/shape features. First-order features are histogram-based features and describe myocardial signal intensity values distribution. Textural features describe the spatial relationship between neighboring voxels signal intensity quantifying myocardial heterogeneity in five matrices: grey level co-occurrence matrix (GLCM), grey level dependence matrix (GLDM), grey level run-length matrix (GLRLM), grey level size zone matrix (GLSZM), and neighborhood grey tone differences matrix (NGTDM). Size/shape features are signal-independent features and describe the 2D- and 3D-geometric properties of the myocardium. All features were normalized to a unity range from zero to one.

**Feature selection and model construction**

The eight feature selection methods including analysis of variance (ANOVA), mutual information (MI), max-relevance and min-redundancy (MRMR), Gini index, eXtreme Gradient Boost (XGBoost), least absolute shrinkage and selection operator (LASSO), support vector machine (SVM) combined with sequential forward selection (SFS), and K-Nearest Neighbor (KNN) combined with SFS. Seven interpretable ML algorithms were investigated: XGBoost, SVM, Lasso, random forest (RF), logistic regression (LR), decision tree (DT), and gradient boosting (GB). These classifiers were employed to develop separate prediction models based on clinical data, radiomics features, and their integration. To ensure robustness and accuracy, we adopted a five‐fold cross‐validation approach for each dataset during the model training phase. After a grid search to optimize the balance between model complexity and predictive accuracy, the XGBoost parameters were set with a learning rate of 0.02, a maximum tree depth of 4, and an ensemble of 600 trees. For the SVM, we selected the radial basis function (RBF) kernel due to its effectiveness with non‐linear data. We meticulously tuned the hyperparameters, optimizing the SVM cost parameter through a grid search within the range [0.1, 1, 10]. Similarly, the gamma parameter for the RBF kernel was fine‐tuned across the values [0.001, 0.01, 0.1]. For LASSO, the regularization strength (α) is tuned via grid search to balance feature selection and accuracy. In the case of the RF classifier, we opted for a 500‐tree configuration, setting the feature count for node splitting to the square root of the overall feature number. Decision Trees are optimized using max_depth, min_samples_split, and leaf size to control overfitting. Gradient Boosting parameters—learning rate, n_estimators, max_depth, and subsample—are jointly tuned to enhance generalization.

**Reference:**

1. Fent GJ, Garg P, Foley JRJ, et al. Synthetic Myocardial Extracellular Volume Fraction. JACC Cardiovasc Imaging. 2017;10(11):1402-1404.

**Table S1. Radiomics quality score**

| **Item** | **Score** |
| --- | --- |
| **Domain 1: Protocol quality and stability in image and segmentation (0 to 5 points)** |  |
| Protocol quality (2) | 1 |
| Test-retest (1) | 1 |
| Phantom study (1) | 1 |
| Multiple segmentation (1) | 1 |
| **Domain 2: Feature selection and validation (-8 to 8 points)** |  |
| Feature selection or adjustment of multiple testing (-3 to 3) | 3 |
| Validation (-5, 2, 3, 4 or 5) | 3 |
| **Domain 3: Biologic/clinical validation and utility (0 to 6 points)** |  |
| Non-radiomic features (1) | 1 |
| Biologic correlates (1) | 0 |
| Comparison to “gold-standard” (2) | 0 |
| Potential clinical utility (2) | 2 |
| **Domain 4: Model performance index (0 to 5 points)** |  |
| Discrimination statistics (2) | 2 |
| Calibration statistics (2) | 2 |
| Cut-off analysis (1) | 0 |
| **Domain 5: High level of evidence (0 to 8 points)** |  |
| Prospective study (7) | 7 |
| Cost-effective analysis (1) | 0 |
| **Domain 6: Open science and data (0 to 4 points)** | 4 |
| **Total** | **28** |

**Table S2. Summary of the number of cardiac magnetic resonance imaging**

|  | **AL-CA patients** | **HCM patients** | **Health control** |
| --- | --- | --- | --- |
| **Development dataset** | 84 | 63 | 34 |
| Native T1 | 84 | 63 | 33 |
| Post-contrast T1 | 78 | 61 | 29 |
| T2 mapping | 67 | 62 | 33 |
| ECV | 78 | 61 | 29 |
| **Validation dataset** | 37 | 21 | 14 |
| Native T1 | 37 | 21 | 14 |
| Post-contrast T1 | 37 | 20 | 14 |
| T2 mapping | 33 | 21 | 14 |
| ECV | 37 | 20 | 14 |

Abbreviation: AL-CA, cardiac light-chain amyloidosis; HCM, hypertrophic cardiomyopathy; ECV, extracellular volume.

**Table S3. Radiomics Feature Extraction**

| **Type of filter** | **Number of extracted features** |
| --- | --- |
| First Order Statistics | 18 |
| Shape-based (2D) | 10 |
| Gray Level Co-occurrence Matrix (GLCM) | 24 |
| Gray Level Run Length Matrix (GLRLM) | 16 |
| Gray Level Size Zone Matrix (GLSZM) | 16 |
| Neighbouring Gray Tone Difference Matrix (NGTDM) | 5 |
| Gray Level Dependence Matrix (GLDM) | 14 |

| **Modal** | **Feature selection** | **Classifier** | **Weighted^*^** |
| --- | --- | --- | --- |
| **Native T1** | MRMR | Random forest | 0.184 |
| **Post-contrast T1** | XGboost | Random forest | 0.208 |
| **T2 mapping** | Lasso | Lasso | 0.194 |
| **ECV** | Lasso | Random forest | 0.211 |
| **Clinical Feature** | ANOVA | XGboost | 0.203 |

**Table S4. The best machine learning models of feature selection and classifier for single-modal data**

Abbreviation: ECV, extracellular volume; MRMR, Max-Relevance and Min-Redundancy.

*The weighted was calculated as the average accuracy of each classifier in 5-fold cross validation, and was used to build the ensemble ML model.

**Table S5. Clinical characteristics, echocardiographic and cardiac magnetic resonance features in the validation cohort**

| **Baseline Characteristics** | **AL-CA patients**  **(n=37)** | **HCM patients**  **(n=21)** | **Health control**  **(n=14)** | ***P* Value** |
| --- | --- | --- | --- | --- |
| **Clinical characteristics** |  |  |  |  |
| Age, years | 68.8±6.2 | 40.5±21.0* | 36.6±13.7** | <0.001 |
| Men, n (%) | 34 (91.9) | 17 (81.0) | 6 (42.9)** | <0.001 |
| SBP, mmHg | 113.8±18.2 | 122.3±19.8 | 123.3±20.9 | 0.174 |
| DBP, mmHg | 69.1±11.0 | 71.3±11.1 | 77.8±14.1** | 0.074 |
| Heart rate, bpm | 84.9±11.6 | 75.4±11.0* | 86.6±15.8 | 0.023 |
| NYHA cardiac function class, n (%) |  |  |  | 0.211 |
| I or II | 30 (81.1) | 20 (95.2) | 13 (92.9) |  |
| III or IV | 7 (18.9) | 1 (4.8) | 1 (7.1) |  |
| Mayo stage, n (%) |  |  |  |  |
| I or II | 18 (48.6) | - | - | - |
| III or IV | 19 (51.4) | - | - |  |
| Hypertension, n (%) | 9 (24.3) | 5 (23.8) | 1 (7.1) | 0.304 |
| Antihypertensive treatment, n (%) | 9 (24.3) | 4 (19.0) | 0 (0) | - |
| Dyslipidemia, n (%) | 3 (8.1) | 2 (9.5) | 0 (0) | - |
| Diabetes mellitus, n (%) | 6 (16.2) | 2 (9.5) | 0 (0) | - |
| Serum creatinine, mg/dl | 81.5±22.0 | 85.4±24.9 | 62.9±13.9** | 0.010 |
| Estimated glomerular filtration rate (mL/min/1.73m2) | 91.6 (72.1-101.6) | 97.7 (59.8-108.4) | 120.4 (115.1-125.9)** | <0.001 |
| cTnI, μg/L | 0.03 (0.01-0.08) | - | - | - |
| NT-proBNP, pg/mL | 1648.5 (257.5-4012.5) | - | - | - |
| dFLC, mg/L | 180.3 (28.3-473.2) | - | - | - |
| **Echocardiography** |  |  |  |  |
| LAD (mm) | 41.8±6.3 | 41.6±7.3 | 31.9±3.8** | <0.001 |
| LVEDD (mm) | 44.3±4.9 | 46.2±4.8 | 45.8±4.1 | 0.378 |
| LVESD (mm) | 29.7±4.4 | 28.9±7.3 | 29.0±4.3 | 0.856 |
| LVPW (mm) | 11.4±3.0 | 10.9±4.4 | 8.2±1.2** | 0.009 |
| RVD (mm) | 22.1±3.4 | 21.3±4.3 | 23.7±7.4 | 0.366 |
| **Cardiovascular magnetic resonance** |  |  |  |  |
| LVEF (%) | 59.2±12.9 | 62.7±14.4 | 62.1±5.4 | 0.857 |
| RVEF (%) | 55.7±11.5 | 64.7±10.0* | 59.1±5.1 | 0.007 |
| LVEDVI (ml/m^2^) | 66.1±16.7 | 80.9±22.1* | 68.0±12.5 | 0.012 |
| RVEDVI (ml/m^2^) | 63.2±18.8 | 62.9±13.8 | 66.3±13.1 | 0.805 |
| IVS, mm | 15.0±3.8 | 21.3±6.0* | 9.8±1.9** | <0.001 |
| LGE positicity(%) | 31 (83.8) | 19 (90.5) | 0 (0)** | <0.001 |

**Abbreviations:** AL-CA, cardiac light-chain amyloidosis; HCM, hypertrophic cardiomyopathy; SBP, systolic blood pressure; DBP, diastolic blood pressure; NYHA, New York Heart Association; cTnI, cardiac troponin I; NT-proBNP, N-terminal pro-B-type natriuretic peptide; dFLC, serum immunoglobulin free light chain difference; LAD, left atrial diameter; LVEDD, left ventricular end-diastolic diameter; LVESD, left ventricular end-systolic diameter; LVPW, left ventricular posterior wall; RVD, right ventricular internal dimension; LVEF, left ventricular ejection fraction; RVEF, right ventricular ejection fraction; LVEDVI, left ventricle end-diastolic volume index; RVEDVI, right ventricle end-diastolic volume index; IVS, interventricular septum; LGE, late gadolinium enhanced.

Data are reported as mean ± SD or number (%) as appropriate.

*P* value indicates for differences across three groups.

^*, **^indicates significant difference (*P* value <0.05) between AL-CA patients and HCM patients, and AL-CA patients and health controls.


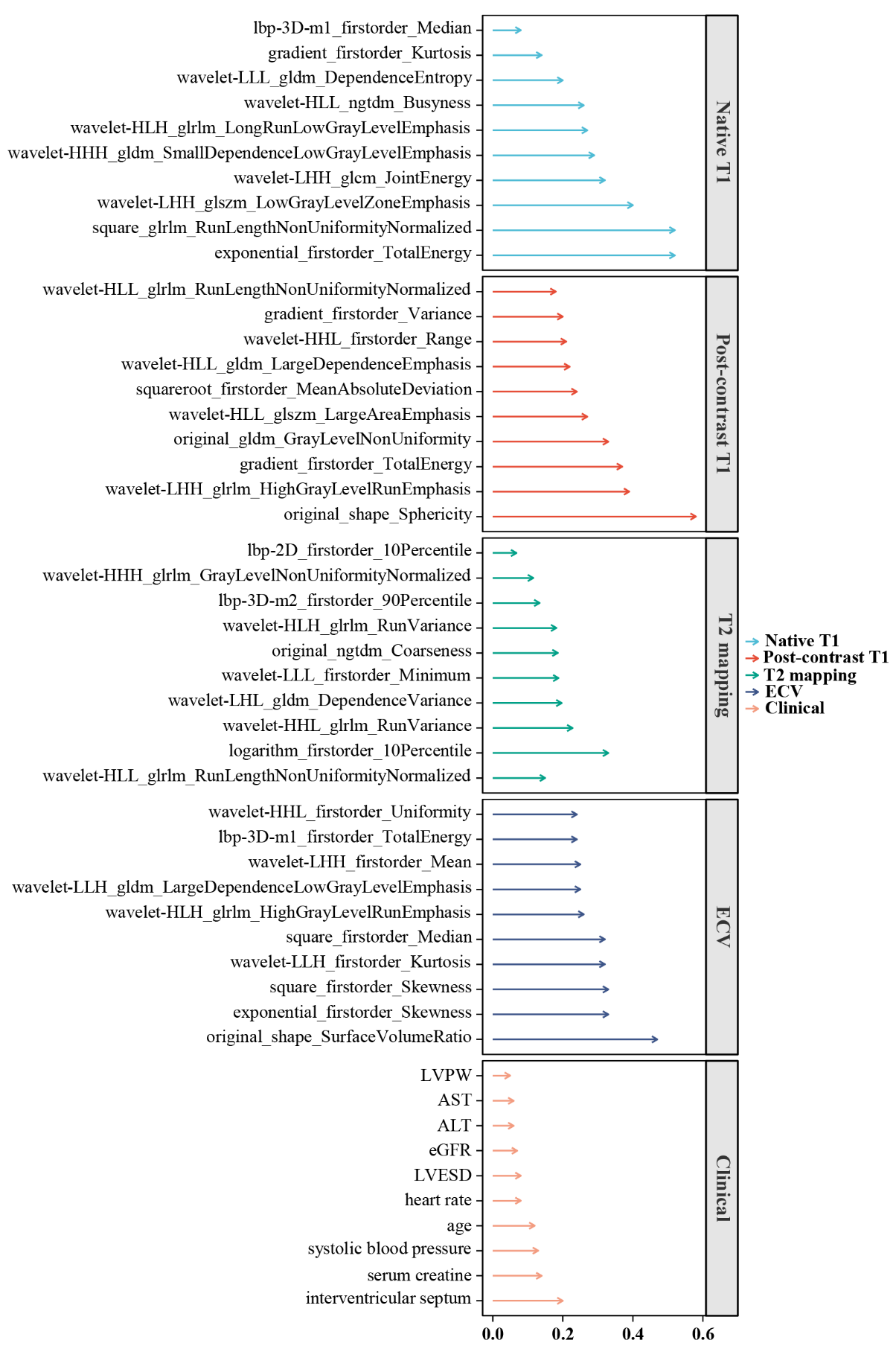
**Figure S1. Variable importance of the top 10 radiomic and clinical features of the ensemble model**


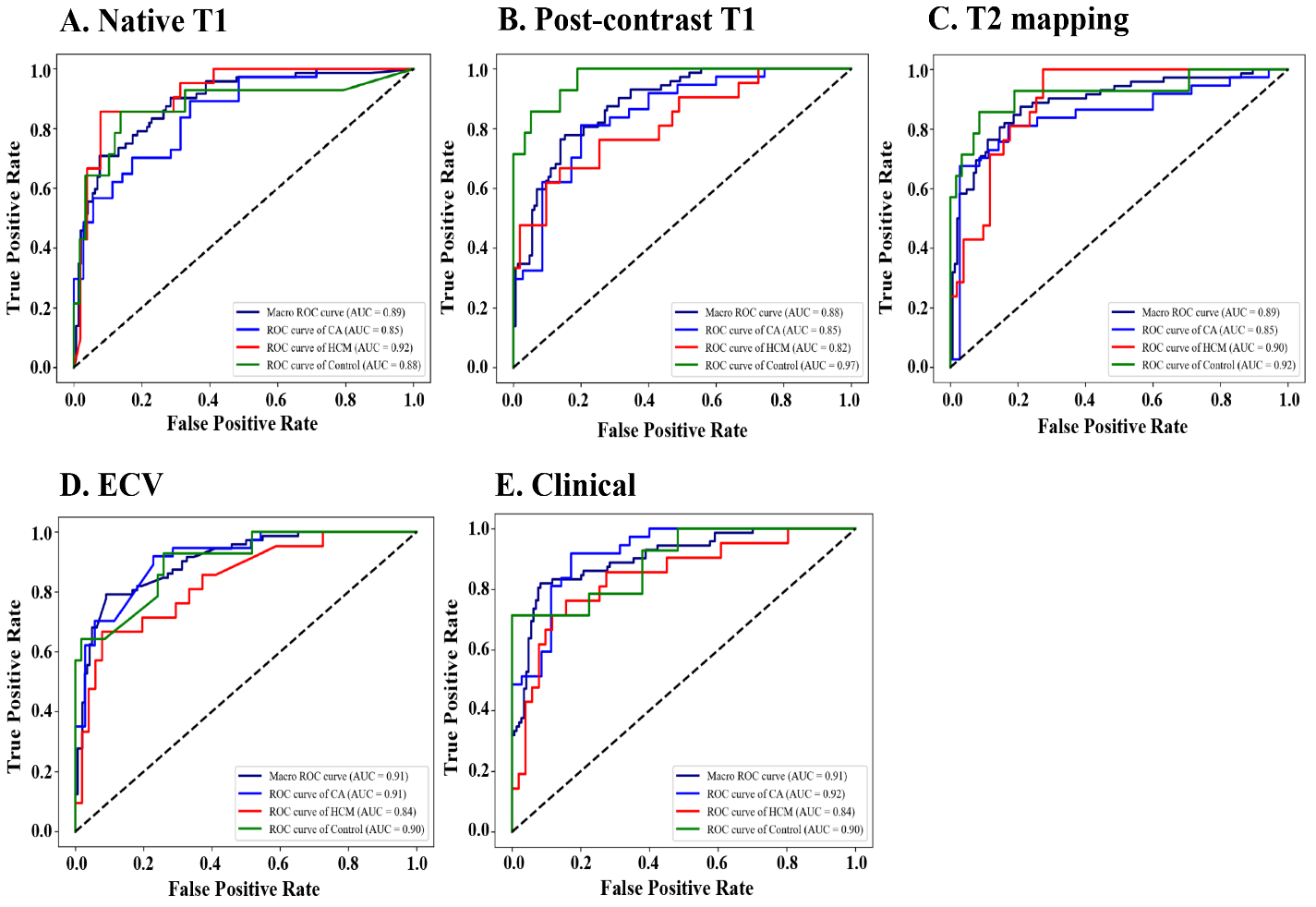
**Figure S2. Performance of single modal classifier for** **detection of cardiac amyloidosis and hypertrophic cardiomyopathy in the hold-out testing set**

Abbreviation: CA, cardiac amyloidosis; HCM, hypertrophic cardiomyopathy; ROC, receiver operating characteristic curve; AUC, area under curve; ECV, extracellular volume.
